# Zebrafish reward mutants reveal novel transcripts mediating the behavioral effects of amphetamine

**DOI:** 10.1186/gb-2009-10-7-r81

**Published:** 2009-07-31

**Authors:** Katharine J Webb, William HJ Norton, Dietrich Trümbach, Annemarie H Meijer, Jovica Ninkovic, Stefanie Topp, Daniel Heck, Carsten Marr, Wolfgang Wurst, Fabian J Theis, Herman P Spaink, Laure Bally-Cuif

**Affiliations:** 1 Department Zebrafish Neurogenetics, Institute of Developmental Genetics, Helmholtz Zentrum Muenchen, German Research Center for Environmental Health, Ingolstaedter Landstrasse, Neuherberg, 85764 Germany; 2 Center for Integrated Protein Science (Munich), Institute of Developmental Genetics, Technical University - Munich, Ingolstaedter Landstrasse, Neuherberg, 85764 Germany; 3 Institute of Developmental Genetics, Helmholtz Zentrum Muenchen, German Research Center for Environmental Health, Ingolstaedter Landstrasse, Neuherberg, 85764 Germany; 4 Institute of Biology, University of Leiden, Leiden, 2300 RA The Netherlands; 5 Institute for Bioinformatics and Systems Biology, Helmholtz Zentrum München, German Research Center for Environmental Health, Ingolstaedter Landstrasse, Neuherberg, 85764 Germany; 6 Current address: Institute of Stem Cell Research, Helmholtz Zentrum Muenchen, German Research Center for Environmental Health, Ingolstaedter Landstrasse, Neuherberg, 85764 Germany

## Abstract

**Background:**

Addiction is a pathological dysregulation of the brain's reward systems, determined by several complex genetic pathways. The conditioned place preference test provides an evaluation of the effects of drugs in animal models, allowing the investigation of substances at a biologically relevant level with respect to reward. Our lab has previously reported the development of a reliable conditioned place preference paradigm for zebrafish. Here, this test was used to isolate a dominant N-ethyl-N-nitrosourea (ENU)-induced mutant, no addiction (*nad*^*dne*3256^), which fails to respond to amphetamine, and which we used as an entry point towards identifying the behaviorally relevant transcriptional response to amphetamine.

**Results:**

Through the combination of microarray experiments comparing the adult brain transcriptome of mutant and wild-type siblings under normal conditions, as well as their response to amphetamine, we identified genes that correlate with the mutants' altered conditioned place preference behavior. In addition to pathways classically involved in reward, this gene set shows a striking enrichment in transcription factor-encoding genes classically involved in brain development, which later appear to be re-used within the adult brain. We selected a subset of them for validation by quantitative PCR and *in situ *hybridization, revealing that specific brain areas responding to the drug through these transcription factors include domains of ongoing adult neurogenesis. Finally, network construction revealed functional connections between several of these genes.

**Conclusions:**

Together, our results identify a new network of coordinated gene regulation that influences or accompanies amphetamine-triggered conditioned place preference behavior and that may underlie the susceptibility to addiction.

## Abstract 2

Transcriptome analysis of a zebrafish mutant that does not respond to amphetamine identifies a network of coordinated gene regulation that may underlie the susceptibility to addiction.

## Background

Addiction, which can be broadly defined as a pathological state characterized by the compulsive seeking and usage of a drug in spite of adverse consequences, is a major societal problem. In the USA alone, more than 23 million Americans are concerned, with societal costs reaching 1.4 million dollars over the life of each addict [1]. Addictive drugs include a large number of substances (such as stimulants, alcohol and opiates) acting through different cellular mechanisms, but which all trigger a sequence of widespread, long-lasting consequences on brain physiology, most of which are only partially understood. The complexity of these plastic events makes it difficult to efficiently care for patients, and current treatments have little power to avoid relapse. As a consequence, a major goal of drug abuse research is to identify the key molecular mechanisms underlying the development of compulsive drug use, which may then be medically targeted for better treatments.

The mechanisms underlying drug addiction utilize a succession of physiological responses that begin with the activation of the brain's reward pathway - a feature common to all drugs of abuse. The reward system, largely based on dopamine signaling projecting to forebrain centers [2], signals a pleasurable experience, which then tends to be repeated. The transition from drug use to addiction [3] occurs gradually and involves both neuro- and synaptic plasticity. These long-lasting adaptive changes persist even after withdrawal of the drug, and they are likely to underlie the persistent tendency to relapse [4]. In addition, several other circuits - in particular the stress axis and the learning and memory circuitry - have been implicated in the reinforcement of drug use or addiction and in the cognitive processes underlying addiction [5]. One powerful approach to understand which molecular alterations contribute to the development and expression of the successive addiction-related behaviors has been the use of microarray expression profiling. Combined with the *in silico *assembly of regulatory networks, this high-throughput analysis can provide a comprehensive picture of the changes in gene expression that may underlie the different steps towards drug addiction. In the case of psychostimulant drugs, for example, microarray analyses have demonstrated the occurrence of important transcriptional changes that differ over time, clearly distinguishing acute from chronic drug use or withdrawal. In models as varied as human post-mortem brains from cocaine abusers and mice or rats of different genetic backgrounds, changes related to molecular pathways controlling neurotransmitter signaling (including a downregulation of the dopamine D2 receptor), signal transduction, ion-gated channel activity, cytoskeletal structures, extracellular matrix remodeling, synaptogenesis, axonal dynamics and cell metabolism [6-8] (reviewed in [9,10]) have been identified.

Because a major step in the development of addiction is the switch from drug use to drug abuse, we aimed to gain insight into the mechanisms triggering the initiation of addictive behavior. Towards this aim, we focused on commonalities in the effects of abused drugs, hence on their early effect on the reward pathway. Based on previous observations demonstrating that the response of the reward system increases with expectancy (thus, it is subject to auto-amplification) [11], we reasoned that a major susceptibility factor in the transition from drug use to abuse might be the intensity of the initial reward response. In order to narrow-down transcriptional approaches to this process, recent analyses compared the transcriptional effects of several drugs [12], or made use of mice carrying alterations in the function of genes postulated to be relevant to reward. For example, the transcriptional effects of cocaine have been compared in mice lacking the dopamine D1 receptor (necessary for the sensitization to cocaine) versus their wild-type siblings [7], in mice overexpressing the immediate early transcription factors CREB or ΔfosB (both of which are involved in mediating the acute effects of cocaine) [13] and in Cdk5 knock-out mice (Cdk5 is a downstream target of ΔfosB) [14].

We aimed to provide an unbiased insight into this question, without *a priori *selection of a regulatory pathway, while remaining clearly associated with the reward behavioral output. To achieve these goals, we initiated a functional study of the reward pathway in zebrafish, a vertebrate model amenable to random mutagenesis and behavioral screening. Reward behavior is an ancestral behavior, conserved throughout vertebrate phyla, and the underlying neurotransmitter pathways are shared between species [15-18]. We chose to use the psychostimulant amphetamine as it directly stimulates the reward pathway (largely via altering the function of the dopamine transporter Dat [19], which elicits limited physical dependency, and on the behavioral assay known as conditioned place preference (CPP)). This test, in which association with the pleasurable effect of a drug modifies an animal's choice for a specific environmental cue, has been classically used as a read-out of the functionality of the reward system [20].

We recently developed a robust assay for amphetamine-induced CPP behavior in adult zebrafish, and demonstrated the role of acetylcholine signaling in the sensitivity to amphetamine-induced reward [17,21]. Here, relying on evidence suggesting genetic components in the susceptibility to addiction (reviewed in [22-24]), we used this assay in a N-ethyl-N-nitrosourea (ENU) mutagenesis screen, successfully isolating an amphetamine-resistant mutant in the CPP test, *no addiction *(*nad*^*dne*3256^; hereafter referred to as *nad*). This mutation is dominant and *nad *heterozygotes fail to change their place preference upon repeated amphetamine administration. In zebrafish, amphetamine does not trigger a locomotor response [17], and lack of CPP is the only phenotype that we could associate with the *nad *mutation to date. We next used this mutant in a three-step expression profiling paradigm comparing the transcriptional response of wild-type animals upon CPP-stimulating amphetamine administration with that of *nad *mutants receiving either drug or a saline control solution. We discovered a set of 139 genes that respond to amphetamine in wild-type animals, but respond inappropriately in *nad *mutants without being altered under basal conditions in either genotype. In addition to genes involved in pathways classically associated with reward, this gene set shows a striking enrichment in transcription factors that are specifically known for their involvement in brain development. We validated a subset of these genes using quantitative PCR (qPCR) and *in situ *hybridization, thereby revealing an association of these gene expressions with neurogenic zones of the adult brain, which is also apparent in the mouse. We developed a database linking zebrafish genes to information on orthologous gene interactions, which we then used to demonstrate that many of these genes contribute to a common regulatory network. Together, our results identify a pattern of coordinate gene regulation that may underlie or accompany the development of CPP behavior upon amphetamine administration and, hence, may contribute to generating a susceptibility background towards the development of addiction.

## Results

### The mutant *nad *fails to respond to amphetamine-induced reward

**Figure 1:**
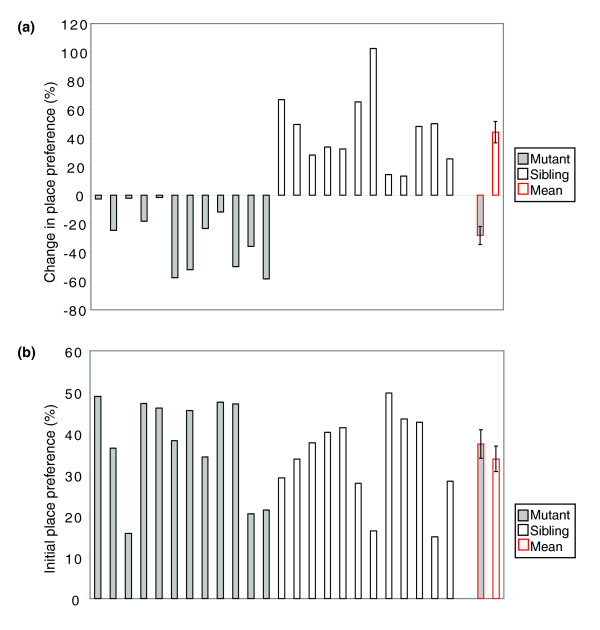
**The dominant mutant *nad*^*dne*3256 ^shows no response to amphetamine, but a normal initial place preference**. **(a) **Conditioned place preference (%) of 24 individuals of a *nad*^*dne*3256 ^family (generation F3), showing 12 mutants and 12 siblings. Mutants were defined as showing no or a negative change in place preference. Siblings were defined as having a change in place preference of 5% or over. The last two bars represent the means for both groups. The difference between the means is statically significant (*t*-test with unequal variances; *P *= 2.3E-07). **(b) **Initial place preference (%) for the same 24 individual fish. The last two bars represent the means for both groups. There is no significant difference between the two means (two sample unequal variance *t*-test *P *= 0.45). Error bars represent the one fold of the standard error.

To recover mutants of the amphetamine response, we designed an ENU mutagenesis screen making use of the amphetamine-based CPP test for adult zebrafish [21]. Briefly, in this test, the psychostimulant amphetamine is associated with the initially non-preferred side of a two-color tank. The repeated administration of amphetamine causes a switch in the place preference of the fish: even in the absence of drug, the animal will now prefer the amphetamine-paired side of the tank. As previously demonstrated using adults heterozygous for the *ache*^*sb*55 ^mutation, this test is robust enough to detect dominant mutations affecting amphetamine-triggered preference [17]. To recover new dominant mutations of this type, we screened F1 animals generated from ENU-treated F0 males for their place preference response to amphetamine. Potential mutants were then out-crossed to wild-type fish and their F2 progeny was retested at adulthood. From 396 F1 animals tested (corresponding to 396 genomes), 4 animals failed to change their place preference upon amphetamine administration while showing normal initial place preference without drug (not shown). One of these potential mutants transmitted this phenotype to 50% of its progeny, following the expected Mendelian distribution for dominant genetic traits (Figure 1a). To date, this transmission has been stable over more than five generations and is detectable equally well in both the AB and the polymorphic AB/Tü background [21], arguing for a *bona fide *dominant mutation. Importantly, the initial place preference in mutants does not differ from that of their siblings (Figure 1b), demonstrating their normal response to the visual cues of the test tank. Following drug treatment, amphetamine brain content is also similar in mutant fish and their siblings (not shown). We named this mutation *no addiction *(*nad*^*dne*3256^).

### A distinct gene expression signature underlies the abnormal behavioral response of *nad *mutants to amphetamine

Previous experiments based on candidate gene or microarray analysis demonstrate that amphetamine treatment has an impact on gene expression (for a review, see [10]). These gene expression changes are likely to mediate or reflect a large part of amphetamine's actions on multiple biological processes, one of which is to activate the reward pathway. The design of our mutant screen further implies that the effect of amphetamine on CPP development is impaired in *nad *(see Discussion for the possible behavioral meanings of *nad*). Thus, the changed transcriptional response of *nad *to the drug can help identify the genes meaningful to the response to amphetamine.

**Figure 2:**
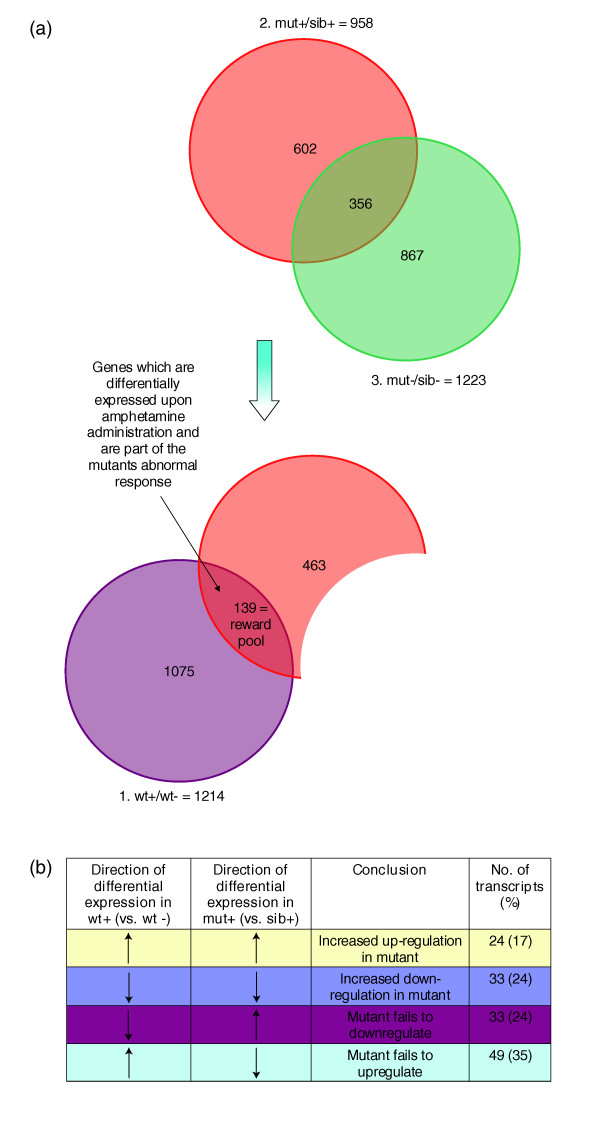
**'Reward pool' genes characterize the transcriptional response to amphetamine-triggered CPP**. **(a) **Diagram of differentially expressed genes from microarray experiments. Individual microarray experiments were combined to reveal a reward pool. A comparison of the differential expression from two experiments showed no bias in the direction of expression. Pool 1 shows the genes differentially expressed in 'wild type with amphetamine versus wild-type without amphetamine'. Pool 2 represents genes differentially expressed in 'mutant with amphetamine versus sibling with amphetamine'. Pool 3 represents genes differentially expressed in 'mutant without amphetamine versus non-mutant siblings without amphetamine'. The genes in pool 3 were subtracted from pool 2 in order to eliminate basal differences between mutants and siblings, not due to amphetamine administration. The intersection of the remaining genes in pool 2 and the genes in pool 1 forms the 'reward pool'. The genes in this pool are differentially expressed in both experiments - that is, they are involved in the wild-type response to amphetamine, as well as the non-response to amphetamine in the mutant. **(b) **Comparison of the direction of regulation (up or down) of transcripts from the reward pool for the experiments wt+/wt- and mut+/sib+. No bias towards a particular pattern can be observed.

We designed three microarray comparisons to specifically isolate such genes (Figure 2a; see Additional data files 1 to 3 for complete gene lists). In a first comparison, we found 1,214 genes to be differentially expressed between wild-type fish that received amphetamine treatment triggering CPP versus fish that received a control, saline treatment (experimental conditions identical to those described above; microarray experiment 1, 'wt+/wt-'; Figure 2a, purple group). To extract genes meaningful to CPP development from this pool, we next identified the transcripts that were differentially affected by amphetamine in *nad *versus their wild-type siblings. Analysis of the microarray data for this second comparison showed that 958 genes were differentially expressed between mutants and wild-type siblings upon amphetamine treatment (experiment 2, 'mut+/sib+'; Figure 2a, pink group). However, as these are likely to include basal transcriptional differences between mutants and wild-type fish (that is, transcriptional differences that are not triggered by amphetamine administration), we performed a third microarray comparison between mutants and their siblings without amphetamine (experiment 3, 'mut-/sib-'; Figure 2a, green group). We found 1,223 genes to be differentially expressed under these conditions, which we then took to represent the basal differences between the mutants and their siblings. Of these, 356 were also differentially expressed in the experiment 'mut+/sib+' and were then subtracted from this group to recover genes characterizing the different response of mutants versus siblings to amphetamine. This subtraction resulted in the pool 'mut+/sib+ minus mut-/sib-'. The intersection of the pools 'mut+/sib+ minus mut-/sib-' and 'wt+/wt-' was taken to form the 'reward pool' - that is, genes that both characterize the wild-type response to amphetamine and that display an altered response (that is, they respond less or more than in wild type) in the mutant with amphetamine, correlating with the failure of CPP in this genotype. This pool comprises 139 genes, which are listed and functionally annotated in Additional data file 4.

Of the 139 genes in the reward pool, 17% were upregulated in both 'mut+/sib+' and 'wt+/wt-' (Figure 2b). Transcription of these genes is increased in wild-type fish upon amphetamine treatment and excessively increased in the mutants. Conversely, 24% of the 139 genes were down-regulated in both experiments; hence, their transcription is normally down-regulated upon amphetamine treatment, and is excessively down-regulated in the mutants. Finally, a majority of the genes (59% of 139) responded to amphetamine in an opposite manner between wild-type and mutant fish (24% of the 139 genes were up-regulated in mut+ compared to sib+, but downregulated in wt+ compared to wt-, and 35% were down-regulated in mut+ compared to sib+ and up-regulated in wt+ compared to wt-). These genes failed to be down- or up-regulated, respectively, in the mutants upon amphetamine treatment.

### The reward pool is significantly enriched in transcription factor-encoding genes

**Figure 3:**
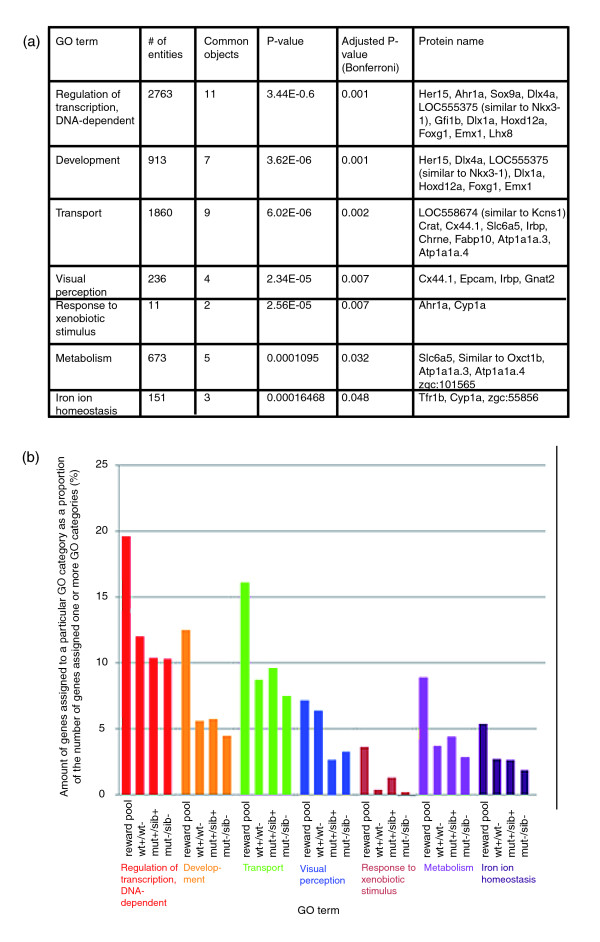
**Categorization of the 139 genes contained in the reward pool**. **(a) **Gene Ontology (GO) term enrichment analysis for terms included in the category 'biological process'. Terms were considered to be significantly enriched if the adjusted *P*-value was < 0.05. Redundant terms have been excluded. **(b) **Bar graph comparing the amount of genes assigned to a particular category as a percentage of all genes that could be assigned one or more GO terms. The depicted categories were chosen from the analysis in (a). For the experiment wt+/wt- 517 genes were assigned a GO term, for mut+/sib+ 386, for mut-/sib- 493 and for the reward pool 56.

In order to further investigate the mechanisms involved in reward, Gene Ontology (GO) enrichment analyses categorizing the genes in the organizing principle 'biological process' were performed on each of the individual experiments and the reward pool (Figure 3; Additional data file 5). We found that the reward pool contains a high proportion of genes encoding functions previously related to reward or the transition to addiction, such as neurotransmitter signaling pathways, ion channels and regulators of neuronal and synaptic plasticity (see Additional data file 4 and Discussion). In order to characterize processes specific to the rewarding effects of amphetamine, we also searched for particular enrichments in the reward pool over the other three gene sets. Enriched categories in the reward pool (Figure 3a) and individual experiments (Additional data file 5) are compared in Figure 3b. The most striking result was that the term 'transcription' was enriched across all groups, but displayed a further relative increase in the reward pool. This was also the case for the term 'development', and, in fact, both superordinate categories largely overlapped in their gene content (see Figure 3a for a list of proteins encoded by these genes). Thus, the involvement of transcription factors previously recognized for their relevance in developmental processes distinguishes the reward response to amphetamine (and its failure in *nad*) over other transcriptional effects of amphetamine treatment.

### Amphetamine-responding genes can be validated by quantitative PCR and classified as acute and/or chronic responders

The proteins encoded by individual genes annotated in the zebrafish genome (Ensembl release zv7 [25] and ZFIN [26]) are listed in Figure 3a corresponding to each term for the reward pool (see Additional data file 5 for the GO term enrichment in the individual array experiments). We chose ten genes for validation based on GO enrichment analysis and literature searches: *ahr1a*, *dlx1a*, *emx1*, *foxg1*, *gfi1b*, *her15*, *lhx8*, *slc6a5*, *sox9a *and *tbr1*. As developmental transcription factors have not been recognized as a signature of the behavioral response to amphetamine in previous studies, our selection was largely biased towards this category: nine of the chosen genes encode transcription factors (*ahr1a*, *dlx1a*, *emx1*, *foxg1*, *gfi1b*, *her15*, *lhx8 *(previously *lhx7*), *sox9a *and *tbr1*), four of which have been assigned the GO term 'development' (*her15*, *foxg1*, *emx1 *and *dlx1a*). In addition to their generally prominent role during brain development, strong arguments to choose these genes were: the maintenance of expression of their orthologues in the adult mammalian brain (respectively in mouse, *Ahr*, *Dlx1*, *Emx1*, *Foxg1*, *Gfi1*, *Hes5*, *Lhx8*, *Sox9 *and *Tbr1*) [27-35]), suggesting an extended role in controlling brain physiology; and their comparable expression patterns in both mouse [32,36-42] and zebrafish [43-48] and our unpublished data), at least during brain development, arguing for conserved functions in these species. In addition, we chose to test *slc6a5 *as a representative of the neurotransmitter pathway genes recovered in the reward pool. *slc6a5 *encodes the glycine neurotransmitter transporter GlyT2, which is involved in the reuptake of glycine at the synapse.

In a first step, expression of these genes in the wild-type adult brain was confirmed using *in situ *hybridization. All ten transcripts gave strong signals in the brain, including the telencephalon (Additional data file 6). Specifically, the expression of *gfi1b *and *her15 *is restricted to the ventricular areas of the telencephalon (Additional data file 6e, f), diencephalon, and midbrain (not shown). *dlx1a*, *emx1*, *foxg1*, *lhx8*, *slc6a5*, *sox9a *and *tbr1 *are expressed in restricted areas of the brain, including subdomains of the pallium and/or subpallium in the telencephalon (Additional data file 6b-d, g-j). Overall, the regional expression of these genes is in keeping with their known expression in the adult mammalian brain (see Discussion). *ahr1a *is expressed ubiquitously throughout the brain (Additional data file 6a; and data not shown).

**Figure 4:**
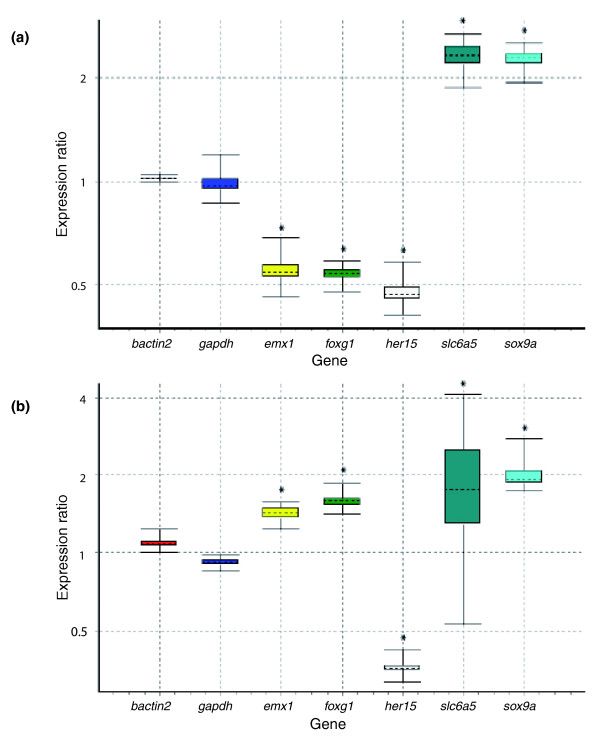
**Validation using quantitative PCR**. Individual genes with different biological roles were selected from the reward pool (Figure 3a) for qPCR using original RNA from **(a) **wt+/wt- and **(b) **mut+/sib+. The qPCR experiment revealed selected genes showed expression changes similar to those seen in the microarray results. The figures show box plots of relative gene expression, where the top and the bottom of each box indicate the 75th and 25th percentiles, respectively, whereas the dotted line represents the median. Asterisks indicate that the probability of the alternative hypothesis (that the difference between sample and control groups is only due to chance (*P*(*H1*)) being correct is ≤ 0.05 (see also Materials and methods).

**Figure 5:**
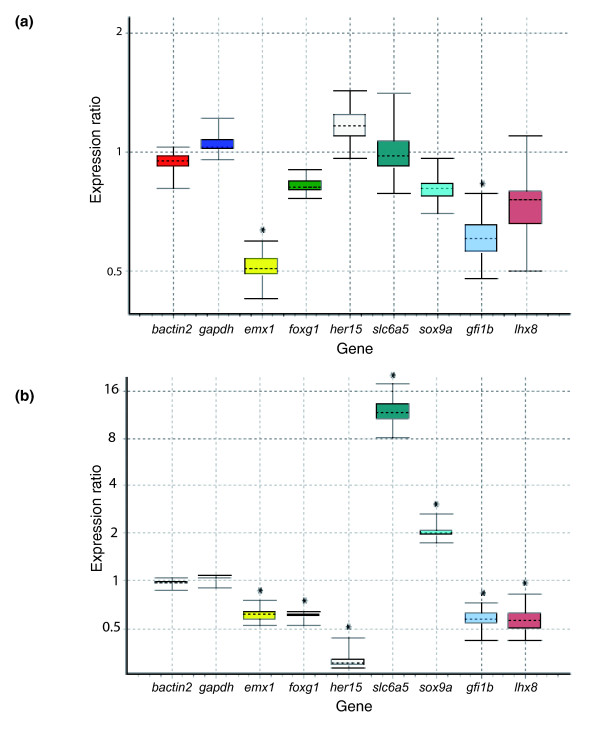
**Validation and categorization of transcripts in acute and/or chronic response to amphetamine using quantitative PCR**. qPCR was performed on the brain of fish injected with one dose (acute) or 18 doses of amphetamine (chronic). **(a) **Two genes, *gfi1b *and *emx1*, were downregulated after one dose of amphetamine. **(b) **The remaining transcripts were down- or up-regulated in the same direction as the microarray in the chronic situation. The figures show box plots of relative gene expression, where the top and the bottom of each box indicate the 75th and 25th percentiles, respectively, whereas the dotted line represents the median. Asterisks indicate that the probability of the alternative hypothesis (that the difference between sample and control groups is only due to chance (*P*(*H1*)) being correct is ≤ 0.05 (see also Materials and methods).

Next, qPCR was used to validate the differential expression of seven of these ten genes upon amphetamine administration (*emx1*, *foxg1*, *gfi1b*, *her15*, *lhx8*, *slc6a5 *and *sox9a*). Five of these genes (*emx1*, *foxg1*, *her15*, *slc6a5 *and *sox9a*) were first re-tested on the original RNA used for the microarrays. All were differentially regulated in the same direction as in the microarray for both wt+/wt- and mut+/sib+, thus validating our microarray experiments (Figure 4a, b; Additional data file 7). We next tested all genes using new RNA samples. Our experimental design for the arrays involved four injections of amphetamine alternating with three doses of saline solution, with the last amphetamine injection given 30 minutes before death. We hypothesized that this would allow us to identify genes reacting to any or just acute or chronic amphetamine administration. As previous studies showed differences in the reaction of transcriptional levels to these different treatments [49], we conducted qPCR experiments on the 7 above-selected genes using brains from fish that had been injected once (acute) or 18 times once daily (chronic) with amphetamine, with the last administration 30 minutes before death. The results are depicted in Figure 5a, b (see also Additional data file 7). *emx1 *and *gfi1b *appeared differentially expressed upon acute amphetamine administration, while there was no difference in *foxg1*, *her15*, *lhx8*, *sox9a *and *slc6a5 *expression between acute-treated and untreated samples. However, all seven genes were differentially expressed upon chronic amphetamine administration, always in the same direction as in the arrays. These results further validate our arrays and, in addition, suggest that the amphetamine administration procedure used to trigger a CPP response in this work is closer to a chronic than to an acute paradigm.

### A subset of the reward pool genes is visibly modulated *in situ *by amphetamine

**Figure 6:**
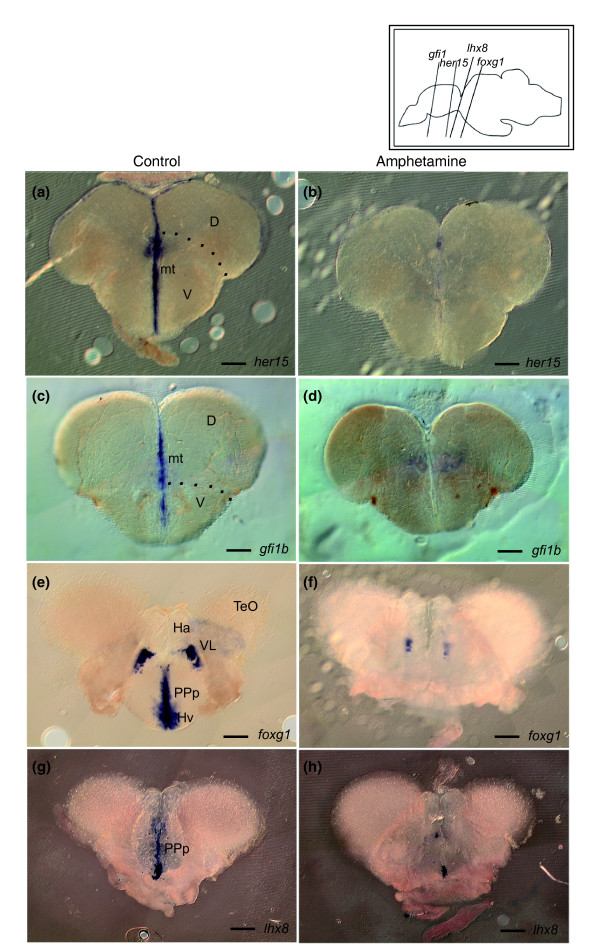
**Candidate genes validated using *in situ *hybridization**. **(a, c) ***gfi1b *and *her15 *are expressed in ventricular zones throughout the brain, including in the midline of the telencephalon. **(b, d) **Upon chronic amphetamine administration, this expression is visibly down-regulated, and this throughout the brain (see also Additional data file 8a-d). Upon amphetamine administration, **(f, h) **the expression of *foxg1 *and *lhx8 *is reduced in the parvocellular preoptic nucleus, posterior part (PPp) (*foxg1 *and *lhx8*) and in the ventral zone of the periventricular hypothalamus (Hv) (*foxg1*), when compared to **(e, g) **the brains of animals injected with a saline solution. The expression pattern of these genes remains unchanged in other brain areas upon amphetamine administration (Additional data file 8e-h). Scale bars = 100 μm in all panels. D = dorsal telencephalic area; Hv = ventral zone of the periventricular hypothalamus; mt = midline of the telencephalon; PPp = parvocellular preoptic nucleus, posterior part; V = ventral telencephalic area; VL = ventrolateral thalamic nucleus.

As demonstrated above, qPCR using total RNA extracted from whole brains was used to validate and extend the results of our microarrays. However, this approach does not provide information as to which regions of the brain are transcriptionally affected by the drug. *In situ *hybridization was next performed on brain sections of fish chronically injected with amphetamine or saline solution (once a day for 18 days). Of the ten genes selected above, the expression of *foxg1*, *gfi1b*, *her15 *and *lhx8 *were visibly changed upon amphetamine administration. The expression of *gfi1b *and *her15*, which characterize the ventricular zone in all brain subdivisions, was completely and consistently down-regulated throughout the brain (Figure 6a-d; Additional data file 8a-d). The expression of *foxg1 *and *lhx8 *were affected in a region-dependent manner. *foxg1 *transcription was reduced in the ventrolateral thalamic nucleus, and eliminated at the midline in the ventral zone of the periventricular hypothalamus and the parvocellular preoptic nucleus - no expression changes were detected throughout the remainder of the brain (Figure 6e, f; Additional data file 8e, f). The expression of *lhx8 *was also much reduced in this latter domain upon amphetamine treatment, but was unchanged elsewhere in the brain (Figure 6g, h; Additional data file 8g, h). The reduction or increase of expression of the other six selected genes, indicated by the array and qPCR data, was not visible using *in situ *hybridization (not shown). As *in situ *hybridization is not a quantitative technique, it is possible that changes in expression must be large before they can be observed using this method. Together, these results highlight that ventricular domains of the adult brain are major areas responding to an amphetamine administration paradigm activating the reward pathway, and identify *gfi1b*, *her15*, *lhx8 *and *foxg1 *as prominent transcriptional targets in these domains.

**Figure 7:**
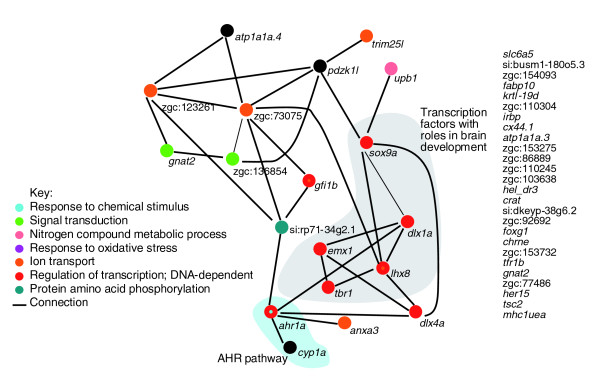
**Network view of 18 genes from the reward pool functionally linked by the ZFISHDB database**. Nodes are connected if functional interactions between the genes are provided by the cluster of orthologous genes (COG) mode of the STRING database. The GO terms listed are not exhaustive. The genes that have a mouse homologue and were thus included in the analysis, but which were not linked to other genes in the pool, are listed separately on the right. In addition, *her15*, *tsc2 *and *mhc1uea*, for which the program did not find suitable mouse homologues, but for which we manually checked for associates with the other genes in the network, are also listed.

We finally aimed to determine whether genes of the reward pool could be functionally connected. We developed a database (ZFISHDB) linking zebrafish genes to functional annotations and relationships via the STRING database. From the 139 genes of the reward pool, 53 could be attributed to a cluster of orthologous genes; 25 interactions were found between 18 of these genes (Figure 7). In particular, eight of the transcription factors, of which five have demonstrated roles in brain development (Dlx1a, Emx1, Lhx8, Sox9a and Tbr1), could be functionally connected, suggesting that amphetamine may re-use a developmental network in mediating reward in the brain.

## Discussion

A major unsolved problem in the field of drug addiction is the characterization of the transcriptional changes underlying the switch from drug use to drug abuse. In this study we used an unbiased paradigm to identify a subset of genes involved in reward activation and its behavioral output. Our approach does not rely on the prior selection of a particular pathway, but rather on a mutant (*nad*) whose only phenotype is the lack of CPP behavioral response to amphetamine. Although we have not yet identified the mutation underlying this phenotype, we were able to extract a subset of 139 genes from the general transcriptional response to amphetamine that respond abnormally to amphetamine in the mutant, correlating with a failure to develop CPP after amphetamine treatment. transcriptional regulation of these genes is, therefore, associated with reward-triggered CPP behavior. We validated the microarray using both qPCR and *in situ *hybridization, thereby identifying neurogenic areas as potentially significant for the response to amphetamine. Our analysis highlighted the predominance of transcription factors in the response to amphetamine. These genes have been recognized for their function during brain development in both zebrafish and mouse, and are also expressed in the adult brain, pointing to the re-use of a developmental network as a potentially important component of reward behavior.

### Behavioral significance of the reward pool

Based on a subset of genes recovered in our array, we used qPCR to show that our experimental conditions mimic chronic amphetamine administration. These genes therefore represent early but not acute transcriptional changes induced by the drug. We do not know, however, whether these changes are long-lasting. Our experimental design also allowed us to focus on a biologically relevant dose of amphetamine with regard to activation of the reward pathway. Finally, the non-response of *nad *mutants suggests that the expression changes recovered are, in part, linked to CPP behavior, although their functional significance with regard to the development of CPP was not addressed in this study. Several parameters underlie CPP behavior and might be altered in *nad*, such as the functionality of the reward pathway itself and the associative learning process involved in CPP, but also changes in tolerance or sensitization to rewarding or motivational events. We have not noticed any other behavioral or morphological alterations in *nad*, and also failed to observe differences in gross neuroanatomy and the organization of several neurotransmitter systems in this mutant (including dopamine and serotonin, revealed by tyrosine hydroxylase and 5-hydroxytryptamine immunocytochemistry, respectively; data not shown). Nevertheless, *nad *animals may exhibit yet other deficient responses to amphetamine that might become apparent if we test later stages of the addiction process, such as the maintenance of drug use, withdrawal or relapse. Whether *nad *mutants are also resistant to other addictive drugs that primarily act through different molecular cascades than psychostimulants, such as opiates [50], and whether the genes of the reward pool are correlatively also transcriptionally modified upon administration of these drugs, remain further very important questions. It will be essential to assess these points in the future to better connect the genes of the reward pool to behavioral function.

### Identification of amphetamine-induced transcriptional changes with no indication of toxic effects

Importantly, we did not find any evidence of genes linked to cell-stress or cell-death, either in individual experiments or in the reward pool. This is in accordance with other microarray expression profiling studies, such as [49], which found little evidence of such genes upon chronic drug treatment. In contrast, many such genes were recovered upon acute administration of psychostimulants (and other drugs like morphine [51]), which may be due to the direct neurotoxic effects of amphetamine or cocaine. Likewise, immediate early transcription factors such as Erg2, Krox24, c-fos, c-jun and CREB, which are often transiently up-regulated following administration of drugs of abuse (reviewed in [9]), were enriched in neither individual experiments nor the reward pool. This confirms that our gene sets reflect transcriptional changes resulting from chronic rather than acute amphetamine action and may mediate the link to the different aspects of addiction. The category of genes related to the biological function 'response to stimulus' was enriched upon drug administration over saline in both wild-type and mutants. However, these genes were filtered out in the reward pool, confirming that they reflect a pharmacological response to the administration of chemical compounds that is unlikely to be altered in *nad *and so might not be involved *per se *in the development of behavioral alterations upon drug taking.

We decided to extract RNA from whole brains, rather than choosing specific anatomical regions. This approach was based on several considerations. Firstly, in addition to acting on the dopaminergic and serotonergic systems, amphetamine raises extracellular levels of glutamate [52] and noradrenaline [53] and these circuits are widely distributed throughout the brain. Secondly, the use of zebrafish makes it difficult to precisely predict where relevant expression changes are to be expected. Although the neurochemical aspects of reward behavior, including CPP, are evolutionarily conserved [18,50,54-56], some of the main neurotransmitter pathways involved in these behaviors show divergent spatial organization between species. For example, the dopaminergic neurons projecting to the zebrafish subpallium (which is hypothesized to be an equivalent of the mammalian basal ganglia, including the nucleus accumbens) are located in the diencephalic posterior tuberculum, unlike in mammals where these neurons lie in the ventral tegmental area of the midbrain [57]. Likewise, the zebrafish brain, as in many other vertebrate classes [58-60], harbors widespread serotonergic clusters as apposed to the single mammalian raphe nucleus [61]. However, as discussed below, we complemented our microarray experiments with *in situ *hybridization in order to allow us to investigate spatial changes in the expression of recovered transcripts and to identify relevant brain areas responding to amphetamine.

### Transcriptionally regulated pathways and reward behavior

Most microarray analyses of reward and addiction to psychostimulants have been conducted in rodents. In addition, one transcriptome analysis of the adult zebrafish brain was recently published, comparing the effects of ethanol and nicotine during withdrawal [54]. It is not possible to analyze all these results side by side given the variety of drugs and drug administration protocols used and in the length of time allowed following drug exposure. Nevertheless, a general outcome was the response to chronic drug use of molecular pathways controlling neurotransmitter signaling (including receptors, transporters and signal transduction components), ion channels and regulators of neuronal activity and plasticity events such as synaptic function or extracellular matrix remodeling [9,13,62-65]. Our manual annotation of the 139 reward pool genes allowed us to identify related mammalian genes in most cases (84 of 139), and to postulate a function based on gene homology or on predicted protein structure for an additional 8 genes (92 of 139), so that our data can be directly compared to previous work. Of the functionally annotated genes of the reward pool, 28 belong to the categories above and 14 have already been linked to reward or addiction (Additional data file 4).

Affected genes related to neurotransmission include those encoding the epsilon subunit of the nicotinic acetylcholine receptor (*chnre*) and glycine transporter 2 (*slc6a5*, formerly *glyT2*), and *LOC793458*, which encodes peptide YYb (PYYb) [66]. All three pathways have been directly or indirectly implicated in reward [67]. We found *chnre *expression to be increased upon amphetamine administration in the wild-type and excessively increased in *nad *animals. Therefore, amphetamine may confer enhanced excitability properties on acetylcholine target neurons via a novel composition of the acetylcholine receptor, which could be linked to the development of the CPP response. Glycine is a major modulator of NMDA receptor-mediated signaling and glutamate neurotransmission is a determining factor in psychostimulant (and other) addictions (for reviews, see [68-70]). It has also been implicated in the regulation of neuronal differentiation, neural network plasticity and synapse dynamics. We found that *slc6a5*/*glyT2 *is increased in wild-type and excessively increased in *nad *animals upon amphetamine administration. Hence, chronic amphetamine administration may modify the amount of glycine at the synapse via Slc6a5 activity, with possible consequences on the development or reinforcement of amphetamine-triggered reward. In mammals, PYY antagonizes the orexigenic and anxiolytic effect of Neuropeptide Y, which can itself elicit CPP reward behavior [71]. We observed *pyy-b *expression to be down-regulated by amphetamine in wild-type but not *nad *animals. Down-regulation of *pyy-b *could reinforce the activity of Neuropeptide Y, thereby contributing to the development of reward, while its lack of response in *nad *animals might mediate the resistance of this mutant to CPP behavior.

Seven genes encoding proteins related to axonal or synaptic dynamics were also recovered in the reward pool (Additional data file 4). Among these, two belong to families that may be directly relevant to addiction or drug use: *Dr. 83111*, encoding a protein highly similar to Neuregulin 1 and a Drebrin-like protein-encoding gene (*Dr. 76820*). Neuregulin 1 signaling plays a prominent role in synapse plasticity in the mature brain by controlling excitatory and inhibitory synaptic transmission [72-74], which might underlie the propensity towards drug abuse [75]. In humans, it has also been identified as a susceptibility factor for schizophrenia, a disease often co-morbid with substance use disorder. We found that Neuregulin 1 was strongly up-regulated by amphetamine in wild-type animals, and massively down-regulated in *nad*. This differential response may play a role in the different CPP behavioral response of *nad*. Drebrin, an F-actin-binding protein enriched in dendritic spines, is essential for spine morphogenesis and activity-dependent synaptic targeting of NMDA receptors [76-79]. Both the morphology and density of dendritic spine in the ventral tegmental area, nucleus accumbens and motor cortex are altered by amphetamine and cocaine [80]. We found that expression of Drebrin is increased upon amphetamine administration in wild-type animals, but fails to be upregulated under the same conditions in *nad *mutants, suggesting that altered changes in dendritic spine remodeling accompany the resistance of *nad *to amphetamine.

Genes encoding components of the dopamine pathway were not identified in our experiment, although amphetamine is considered to primarily increase extracellular dopamine levels in the forebrain [81,82]. In drug-addicted subjects, the concentration of dopamine receptors (namely D2) is lowered on the cell surface as revealed by imaging studies [83]. However, corresponding changes in gene expression have not been consistently reported, suggesting that the modulation of the dopamine pathway may not occur at the level of transcription [9]. Alternatively, microarray sensitivity may be insufficient to detect functionally relevant but small amplitude changes in the expression of weakly expressed factors such as dopamine signaling components [10]. In support of this, although our microarray chips contained a large representation of genes encoding transporters, receptors, and synthesis and metabolism enzymes for most neurotransmitters (including dopamine, glutamate, noradrenaline, 5-hydroxytryptamine, Neuropeptide Y, acetylcholine, glycine and opiates), we only obtained reproducible hybridization, indicating sufficient expression, for a few of these genes (Additional data files 1 to 3). From these, apart from *chnre *and *slc6a5 *(discussed above), only *glutamate transporter 5A *and *neuropeptide Y receptor Y7 *showed significantly modified expression upon chronic amphetamine exposure (an up-regulation in both cases). However, because similar changes were observed in *nad *mutants, these two genes were not present in the reward pool and so are unlikely to account for the non-development of CPP in *nad*.

### *nad *mutants highlight the importance of brain developmental transcription factors in the CPP response to amphetamine, and point to a link between amphetamine administration and the control of adult neurogenesis

An exciting new contribution of our work is to highlight the importance of transcription factors implicated in development in the response to amphetamine-triggered reward. The category 'transcription' was further enriched in the reward pool over individual experiments and so was prominently revealed by our combined microarray strategy. The significance of transcription factors is further strengthened in that all genes classified under 'development' in this analysis are also transcription factors (Figure 3b), and is supported by several validations. Firstly, all chosen transcription factors of the reward pool tested by qPCR (n = 6) displayed changes in transcription levels upon amphetamine administration in wild-type animals and, in four cases, these changes were severe enough to be detected by *in situ *hybridization. The altered response of these four genes upon drug treatment in mutants compared to wild-type was also validated by qPCR. Secondly, several of these genes (*Ahr1*, *Dlx1*, *Foxg1*, *Hes5*, *Sox9 *and *Tbr1*) have been related to drug use or administration in mammals in other studies [62,67,84-87]. Finally, these genes appear to be functionally connected according to the ZFISHDB software; thus, they may participate in a common regulatory network. Strikingly, all these genes have recognized roles during vertebrate brain development and also display persistent expression in the adult brain (Additional data file 6), including the mouse brain (see below), suggesting that their relevance for reward-induced behavior could be extended to adult mammals.

Together, these observations suggest that the development of CPP behavior may rely on the regulation of developmental genes that possibly maintain a modulatory role during adulthood, perhaps contributing to brain plasticity. Brain plasticity is thought to contribute to the learning of addictive behaviors and can underlie long-lasting changes mediating the persistent effects of addiction [88], and several recent reports show that embryonic factors can be recycled in the adult to regulate brain plasticity (for example, [89]). Thus, it is possible that the transcription factors identified in our reward pool are normally reused for modulatory or adaptive processes in the adult, and would here contribute to plasticity events triggered by the drug during the development of CPP. It will be crucial to test the function of these factors in the adult brain, also in the light of behavioral assays, to understand the functional relation between their functional regulation and the stepwise development of addiction behavior.

Classically, the modulatory events believed to underlie addiction involve synaptic or signaling plasticity. The gene regulation network we uncovered can also serve as a valuable entry point towards identifying further plasticity process(es) that might underlie the different behavioral effects of amphetamine in wild-type animals versus *nad *mutants. Our results highlighting developmental transcription factors suggest that fundamental cellular reconfigurations might also contribute to plasticity. In addition, expression of these factors in the mouse and fish brain, and functional assessments in mouse, all point towards a prominent role during neurogenesis. *dlx1a*/*Dlx1 *is expressed in the developing mouse ventral forebrain where it controls the formation of GABAergic neurons [36]. In the adult brain, it is involved in maintaining hippocampal interneurons [30]. *Emx1 *participates in the regionalization of the embryonic mouse cortex and the production of neuronal subtypes [37,38], and adult mice mutant for *Emx1 *exhibit impaired hippocampal neurogenesis [29]. Mouse *Lhx8 *is required for the development and maintenance of forebrain cholinergic neurons [32,39]. Mouse *Sox9 *is present in the stem cells of the peripheral and central nervous system, is essential for gliogenesis [90] and has also been isolated as a co-factor for proneural genes [91]. *Tbr1 *expression characterizes a freshly postmitotic state in the formation of glutamatergic pyramidal projection neurons of the developing mouse neocortex [41], and is maintained during adult hippocampal neurogenesis [33]. AhR (aryl hydrocarbon receptor) overexpression in developing neurons has been linked to premature differentiation [92]. Finally, although Her15 (and its mouse orthologue Hes5) and Foxg1 have not been connected to other transcription factors based on the literature co-citations used by our database, both genes are also expressed in embryonic neuroepithelial progenitors where they are involved in progenitor maintenance [93-96]. Later, *FoxG1 *is strongly expressed in areas of adult neurogenesis, including the subependymal zone of the lateral ventricle and the dentate gyrus of the hippocampus, and juvenile mice haploinsufficient for *FoxG1 *show impaired hippocampal neurogenesis [27]. *Hes5 *expression has also been described in astrocytes in neurogenic zones of the adult mouse brain [28]. Together, these data suggest that most of the transcription factors recovered in the reward pool are linked by their function or at least their expression at one or the other step of neurogenesis control, including in the adult brain. Although we do not yet have a complete account of zebrafish gene expression patterns at the single cell level, our observations are in agreement with this hypothesis in adult fish as well: all the transcription factor-encoding genes investigated in this study have in common that they are expressed in all or part of the adult forebrain ventricular zone (Additional data file 6), which has been demonstrated to be neurogenic [97-101]. The expression profiles of *her15 *and *gfi1b *are particularly striking due to their strict restriction to the ventricular zone (Additional data file 6e, f) and their massive down-regulation upon chronic amphetamine treatment (Figure 5). *emx1*, *sox9a *and *tbr1 *are also noteworthy for their prominent expression in the neurogenic area of the lateral pallium (Additional data file 6c, I, arrows; and data not shown), an area thought to be the functional equivalent of the hippocampus [102,103].

A link between adult neurogenesis and drug abuse has been previously investigated, although with mixed results. Overall, the effect of amphetamine on proliferation during chronic application remains to be examined, although chronic cocaine use has been shown to decrease cell proliferation in the germinal zone of the adult mouse hippocampus [104,105] (for reviews, see [105,106]), while withdrawal from cocaine self-administration triggers accelerated maturation of adult newborn hippocampal neurons [107]. Given the postulated function of adult hippocampal neurogenesis in the acquisition and consolidation of memories (including their spatial and contextual components), these alterations could play a role in the cognitive processes associated with the development, reinforcement or relapse of addiction. Our results strongly suggest that amphetamine also triggers changes in adult neurogenesis (this paper, and KJW, unpublished observations), which might involve or result in the changes in transcription factor expression that we observed. It is now important to investigate this point in detail. Our experimental strategy relying on the lack of behavioral response of *nad *mutants further stresses that the regulation of these transcription factors might directly or indirectly link amphetamine and behavior. However, it seems unlikely that the development of CPP observed after 7 days, and which fails in *nad *mutants, could already result from an effect of amphetamine on adult neurogenesis. Newborn neurons require at least 3 weeks to be incorporated into active circuits in the adult mouse and our previous data suggest a similar time-frame in zebrafish [97]. It is possible, however, that rapid alterations of the ventricular zone by amphetamine could indirectly affect the physiology of neurons in the vicinity, for instance by altering the trophic support normally provided by ventricular radial glia cells leading to an effect on CPP. These changes could be modified in the *nad *mutant. Alternatively, modified neurogenesis upon amphetamine administration could account for later behavioral changes, a hypothesis that remains to be tested in our mutants.

## Conclusions

Our experimental strategy based on the *nad *mutant, which fails to respond to amphetamine in the CPP test, allowed the first identification of a subset of amphetamine-regulated transcripts linked to the reward response. This pool contains gene categories previously linked to the use of addictive drugs, thereby validating our data. Enrichment analyses, confirmed by qPCR and *in situ *hybridization, highlighted a set of genes encoding transcription factors within this pool, most of which are involved in brain development, and which can partially be organized into a network of functional interactions. Together, we propose that the re-use of a developmental transcription factor-mediated network accompanies or underlies the behavioral response to amphetamine in the adult brain. Some of these factors, expressed in adult neurogenic domains and dramatically down-regulated by amphetamine, can further serve as valuable new entry points into studying the link between neurogenesis and addiction.

## Materials and methods

### Animals and maintenance

Adult zebrafish were kept in the fish facility as described in Kimmel *et al*. [108]. For practical reasons (ease of intraperitoneal injections) all experiments were performed on females. In preliminary experiments, we did not notice any difference in the response of males and females to D-amphetamine for a given genotype [21]. Throughout the experiment, care was taken to perform procedures involving animals, such as place preference measurements and injections, at the same time of the day. Mutagenesis, mutant screening and array experiments involving mutants and siblings were performed on fish of the AB background. Mutant fish were maintained in this background throughout the study. Behavioral experiments on wild-type fish were conducted on an intercross background between AB and Tübingen (Tü). The AB and Tü lines are both wild-type lines. AB fish have been inbred over more than 100 generations to date [26], and Tü fish over approximately 25 generations. Both lines, as well as their common progeny (AB/Tü), are sensitive to amphetamine with a similar dose-response and perform equally well in the CPP test [21]. They have been used here for reasons of availability.

### ENU mutagenesis and screening for dominant mutations affecting reward

Adult males of the AB strain were subject, over a 4-week interval, to four 1-hour incubations in 3 mM ENU. Three weeks after this treatment, F1 animals were generated by pair-wise mating of ENU mutagenized males with AB females. The specific locus rate at this stage was estimated to be 1/670 against the *golden *(*slc24a5*) locus. Three- to nine-month-old F1 animals were screened for their change in place preference in response to 40 μg/g D-amphetamine (throughout the text referred to as amphetamine), as described below. Of the 396 F1 adults that were screened, 4 failed to respond to amphetamine, although they exhibited normal place preference without drug and, hence, could recognize the visual cues of the test tank. They also displayed normal amphetamine content in the brain after injection, as measured using denaturing high-performance liquid chromatography (not shown). These animals were considered potential dominant amphetamine-resistant mutants and were crossed against wild-type AB fish to test for transmission of the phenotype. The behavior of 20 F2 adults from these crosses was again assessed in the CPP test in response to amphetamine. For one of these four F1 candidate mutants, 50% non-responders were obtained in the F2 and all further generations, arguing for a *bona fide *dominant mutation. We refer to this mutation as *nad*^*dne*3256^.

### Behavioral assays

The CPP experiment was performed according to Ninkovic and Bally-Cuif [21]. Briefly, the fish were habituated to a biased two-part chamber (days 1 to 2), followed by the determination of the initial place preference (day 3). Subsequently, for test animals, amphetamine injections (40 μM; days 4, 6 and 8) were paired with the initially non-preferred side of the chamber, and control injections of saline solution (days 5 and 7) were paired with the initially preferred side. Control animals are injected with saline every day but likewise paired with the initially non-preferred side on days 4, 6 and 8 and with the initially preferred side on days 5 and 7. On day 9 the final place preference was measured. Conditioning was estimated as in Ninkovic and Bally-Cuif [21] as the change in place preference before and after treatment, relative to the place preference before treatment. Within a mutant family, fish were designated as mutant (mut) when there was no change or a negative change in place preference after amphetamine administration. Fish were designated as wild-type siblings (sib) when the percentage of change was higher than 5%. If the percentage of change was between 0 and 5%, the fish were not included in the microarray analysis in order to avoid incorrect phenotyping.

### Microarray study design and data analysis

The experimental design of this study was aimed at the identification of genes that respond differently to amphetamine treatment in wild-type zebrafish and the reward mutant *nad*. In order to identify the signature gene set for the interaction term genotype*amphetamine treatment, we examined three biological contrasts: the response of wild type (wt) on amphetamine (experiment 1); the differential gene expression in the presence of amphetamine between wild type and mutant (experiment 2); and the base line difference in transcription between wild type and mutant (experiment 3). The animals used in the different experiments were all aged between 6 and 12 months and were manipulated as follows. Experiment 1: wt+, AB fish subjected to the CPP behavioral assay and sacrificed 30 minutes after the final amphetamine injection (the day after place preference determination; day 10); wt-, AB control fish of the CPP behavioral assay (with control saline injections at the same time points as the wt+ fish were injected with amphetamine). Experiment 2: mut+, AB animals from a *nad *family of the F6 generation (obtained from pairing a *nad*/+ F5 heterozygote fish and an AB fish) identified as mutant based on the CPP assay and sacrificed 30 minutes after the final amphetamine injection on day 10; mut-, siblings identified as wild type in the same experiment, and treated exactly the same as the mut+ fish with regard to amphetamine administration. Experiment 3: mut-, AB animals from a F5 *nad *family identified as mutant in the CPP test and left without drug for 2 months afterwards; sib-, siblings identified as wild type in the same experiment and left without drug for 2 months.

One-color Agilent microarray experiments were performed using three biological replicates for each condition. Each replicate contained the RNA from four to five pooled brains. Microarray data were imported into Rosetta Resolver 7.1 (Rosetta Biosoftware, Seattle, Washington, USA) and analyzed using the Rosetta error model for gene expression analysis. The Resolver system calculates ratios for the Agilent intensity microarrays by combining all pairwise ratios of the individual sequence data making up the numerator (for example, treatment replicates) against those making up the denominator (for example, the control replicates) of the specified ratio. The calculations begin with scaling the intensity signals for each sequence relative to the average intensity signal of the entire array. For each sequence, the two scaled intensity values for each pairwise ratio are then converted to the logarithmic scale and the averaging and ratio computation is performed on the logarithms. The sequence errors are accordingly propagated through the log-transformation and averaging. This propagated error is used to determine the statistical significance of the final logarithm of the ratios, that is, the *P*-values corresponding to differential expression are calculated based on the log(ratio). In the Rosetta analysis, first our microarray data were subjected to default intensity error modeling and results from triplicate experiments were combined using the default intensity experiment builder. Next, ratio experiments were built from the intensity data using the Agilent/Intensity-pairwise ratio builder with the control group (salt for experiment 1 and wild type for experiments 2 and 3) as baseline. Data were analyzed at the level of UniGene clusters (UniGene build #105). The resulting *P*-values from the Rosetta error model for gene expression analysis were adjusted for multiple testing using the method of Benjamini and Hochberg to control the false discovery rate. *P*-values were corrected with the p.adjust function in R statistical software. The significance cut-offs were set at *P *< 0.01 (Benjamini and Hochberg adjusted *P *< 0.1) and absolute fold change > 1.5. The false discovery rates at the initial *P*-value cut-off of < 0.01 are, for each experiment: experiment 1, < 0.0816; experiment 2, < 0.07302; experiment 3, < 0.0504. Venn diagrams were constructed using the 'compare biosets' function of Rosetta resolver.

### RNA extraction and microarray study design

Total RNA was extracted from whole brains using RNeasy Mini Kit (Qiagen GmbH, Hilden, Germany) following the protocol 'Purification of Total RNA from Animal Tissues'. For the disruption and homogenization step, brains were dissected and immediately frozen in liquid nitrogen. Then, 600 μl buffer RLT was added to each brain and the tissue was homogenized using a needle and syringe. The samples were individually controlled for RNA quality and genomic contamination using 2100 Bioanalyzer (Agilent Technologies, Palo Alto, CA, USA), according to the manufacturer's instructions. Samples from four to five brains were then pooled to generate a single replicate. The animals used in the different experiments were all aged between 6 and 12 months.

### RNA amplification, labeling and hybridization

The RNA samples were amplified with the Agilent Low Input Linear Amplification kit PLUS, One Color (Agilent Technologies). The labeling, hybridization and data extraction were performed at ServiceXS (Leiden, The Netherlands). Briefly, 500 ng total RNA in an 8.3 μl volume was mixed with 1.2 μl of T7 promoter primer. Primer and template were denatured by incubating at 65°C for 10 minutes and annealed by placing the reaction on ice. The first strand reaction was performed by adding a master mix containing 5× First Strand Buffer, dithiothreitol, 10 mM dNTP mix, RNaseOUT, and Moloney murine leukemia virus reverse transcriptase, and incubated at 40°C for 2 hours. The Moloney murine leukemia virus reverse transcriptase was inactivated by incubation at 65°C for 15 minutes and the samples were directly transferred to ice. Samples were labeled by adding 2.4 μl cyanine 3-CTP. *In vitro *transcription was initiated by addition of the IVT Mastermix containing 4× transcription buffer, dithiothreitol, NTP mix, 50% polyethylene glycol, RNaseOUT, inorganic pyrophosphates, T7 RNA polymerase and incubated at 40°C for 2 hours. Qiagen RNeasy mini spin columns were used for purification of the labeled cRNA as described in the Agilent user manual. After amplification and purification, the samples were checked for RNA concentration and dye incorporation on the Nanodrop ND-1000 by using 1 μl of the 60 μl elution solution (nuclease-free water). Hybridization and washing was performed using the standard Agilent protocol.

The microarray slides were custom designed by Agilent Technologies. The slides contained, in total, 43,371 probes each 60 oligonucleotides long. Of these probes, a total of 21,496 were identical to the probes present on the Agilent probe set that is commercially available under catalog number 013223_D. Most of the additional probes were designed using the eArray software from Agilent Technologies [109]. Settings used were the following: base composition methodology, best probe methodology and design with 3' bias. The Agilent *Danio rerio *transcriptome was used as a reference database. A small number of probes were manually designed in order to obtain gene-specific probes for members of larger gene families. The complete design of the microarrays has been submitted to the Gene Expression Omnibus (GEO) database, under the platform submission number [GEO:GPL7735].

### Microarray imaging and data analysis

Scanning of the microarray slides was performed using the Agilent dual laser DNA microarray scanner. The microarray data were processed from raw data image files with Feature Extraction Software version 9.1, protocol GE1-v1_91(Agilent Technologies). The microarray slides were custom designed by Agilent Technologies as described in Stockhammer *et al*. [110]. Unigene lists of individual experiments can be found in Additional data files 1 to 3. All microarray data were submitted to the GEO database [GEO:GSE14399].

### Gene Ontology term enrichment analysis

GO term enrichment analysis was performed on differentially expressed genes (*P *< 0.01; fold change <-1.5 or > 1.5) from the individual experiments, as well as on the reward pool. Lists of differentially expressed genes were imported into Pathway Studio (Ariadne Genomics, Rockville, MD, USA). Before analysis the ResNet 5.0 database of this software was extended to include the zebrafish protein annotation. The Pathway Studio program determines the human, rat and mouse orthologues of the zebrafish transcripts, using the BLAST best reciprocal hit method. This information is used to perform the enrichment analysis, in which Pathway Studio calculates the statistical significance of the overlap between the input list and a GO group by applying Fisher's exact test. The resulting *P*-value depends on the extent of overlap between the input list and a group as well as the sizes of the list and a group. In addition, we performed a Bonferroni correction for *P*-values calculated from Fisher's exact test by using the p.adjust function in the package 'stats' of the statistical software R [111]. We considered a GO term to be statistically significant if the corresponding adjusted *P*-value is < 0.05.

### Assessment of the functional interactions between recovered genes

Functional interactions between zebrafish genes in the reward pool were inferred from the STRING database [112]. STRING integrates and scores information derived from high-throughput experiments, genomic context, and previous knowledge such as text-mining of abstracts. For zebrafish, the number of interactions is small compared to better annotated species like mouse or human. In order to enrich the interactions in our gene set, we transferred interactions from orthologous genes as provided by the cluster of orthologous genes mode of STRING, where the information of orthologous groups of proteins relies on an extended version of the COG database [113]. The ZebraFish Interaction SearcH DataBase (ZFISHDB) integrates all interactions between clusters of orthologous genes, relying on STRING 7.0. It allows the input of a set of fish genes and outputs interactions between those genes with a STRING combined score above 0.8. In addition, ZFISHDB offers a gene ontology filter to reduce the size of large data sets. It is publicly available [114]. Genes without mouse homologues are not considered by the present version of the database.

### Quantitative real-time PCR

Total RNA was extracted from whole brains using the RNeasy Mini Kit (Qiagen). The qPCR experiments and the statistical analysis were performed using the LightCycler 1.2 system (Roche, Basel, Switzerland) and the relative expression software tool REST [115] as previously described [116]. Briefly, the statistical model used by this software is a pair-wise fixed reallocation randomization test. The software returns the probability of the alternative hypothesis (*P*(*H1*)), which is that the difference between sample and control groups is due only to chance. For each test 50,000 randomization iterations were applied and an associated probability (*P*-value) of *P *< 0.05 was considered significant. Real time PCR experiments were performed in replicates of eight. The list of used primers and probes is provided in Additional data file 9.

### *In situ *hybridization

*In situ *hybridization was performed on 5- to 6-month-old AB/Tü fish that had either been treated with amphetamine (40 μM) or saline solution, once a day, for 18 days. Animals were sacrificed and the brains were removed after fixation in 4% paraformaldehyde. Dissected brains were then postfixed in 4% paraformaldehyde overnight. The brains were then embedded in albumin-gelatine:sucrose denatured with glutaraldehyde. Cross-sections of 70 μm were made using a vibratome, after which the sections were washed in PBS-0.1% Tween-20 and dehydrated through a methanol series. *In situ *hybridization was performed according to published protocols [117] for whole-mount embryos, followed by staining for alkaline phosphatase activity using Nitro-Blue Tetrazolium (NBT)- 5-Bromo-4-Chloro-3-Indolyl-Phosphate (BCIP) (Roche, Basel, Switzerland). Initially, one brain was used per treatment. All sections were photographed and corresponding sections were compared between treatments. The *in situ *hybridization for genes, the expression patterns of which showed a visible difference between amphetamine treatment and control, was repeated once. Sections were photographed with an Axioplan2 stereomicroscope and processed using the Axiovision 4.1 software (Zeiss). To generate probes, partial cDNAs for the genes of interest were cloned from PCR products (PCR conditions available upon request; PCR primers provided in Additional data file 10) into pCRII-TOPO (Invitrogen, Karlsruhe, Germany); for *her15*) or pSC-A-amp/kan using the StrataClone PCR Cloning Kit (Stratagene, La Jolla, CA, USA); for all other genes) following the manufacturers' instructions. All clones were verified by sequencing. The RNA probes were synthesized following published protocols [117].

## Abbreviations

CPP: conditioned place preference; ENU: N-ethyl-N-nitrosourea; GEO: Gene Expression Omnibus; GO: Gene Ontology; NBT: Nitro-Blue Tetrazolium; qPCR: quantitative PCR.

## Authors' contributions

KJW designed and performed the experiments and wrote the manuscript (with LB-C), WN performed mutant identification experiments and worked on the manuscript, JN conducted the behavior screen and identified the *nad *mutant, ST provided technical assistance for *in situ *hybridizations, DT performed the Pathway Studio analyses in WW's lab, AHM and HPS designed the arrays and helped with their interpretation, DH, CM and FT designed the ZFISHDB database, and LB-C directed the work, designed the experiments and wrote the manuscript (with KJW).

## Additional data files

The following additional data are available with the online version of this paper: a complete Unigene list of genes recovered in microarray experiment 1 'wt+/wt-' (Additional data file 1); a complete Unigene list of genes recovered in microarray experiment 2 'mut+/sib+' (Additional data file 2); a complete Unigene list of genes recovered in microarray experiment 3 'mut-/sib-' (Additional data file 3); a list of the 139 genes contained in the reward pool (Additional data file 4); a list of enriched GO terms in individual experiments (Additional data file 5); figures showing expression in the adult telencephalon for ten transcripts chosen for validation (Additional data file 6); qPCR data for validated transcripts in conditions of chronic and acute amphetamine administration (Additional data file 7); figures showing expression of *her15*, *gfi1b*, *foxg1 *and *lhx8 *in the adult brain upon amphetamine administration (Additional data file 8); a list of the primers and probes used for qPCR (Additional data file 9); a list of the primers used to clone *in situ *hybridization probes (Additional data file 10).

## Supplementary Material

Additional data file 1 Complete Unigene list of genes recovered in microarray experiment 1 'wt+/wt-'. Click here for file

Additional data file 2 Complete Unigene list of genes recovered in microarray experiment 2 'mut+/sib+'. Click here for file

Additional data file 3 Complete Unigene list of genes recovered in microarray experiment 3 'mut-/sib-'. Click here for file

Additional data file 4 The 139 genes contained in the reward pool. Click here for file

Additional data file 5 Enriched GO terms in individual experiments. Click here for file

Additional data file 6 Expression in the adult telencephalon for ten transcripts chosen for validation. Click here for file

Additional data file 7 qPCR data for validated transcripts in conditions of chronic and acute amphetamine administration. Click here for file

Additional data file 8 Expression of *her15*, *gfi1b*, *foxg1 *and *lhx8 *in the adult brain upon amphetamine administration. Click here for file

Additional data file 9 Primers and probes used for qPCR. Click here for file

Additional data file 10 Primers used to clone *in situ *hybridization probes. Click here for file
