# Supplementary material for: Zebrafish reward mutants reveal novel transcripts mediating the behavioral effects of amphetamine
Source: Genome Biol. 2009 Jul 31;10(7):R81. doi: 10.1186/gb-2009-10-7-r81 (PMC2728535; doi:10.1186/gb-2009-10-7-r81)
Supplement: Additional data file 6 — Expression in the adult telencephalon for ten transcripts chosen for validation. [file gb-2009-10-7-r81-S6.pdf]

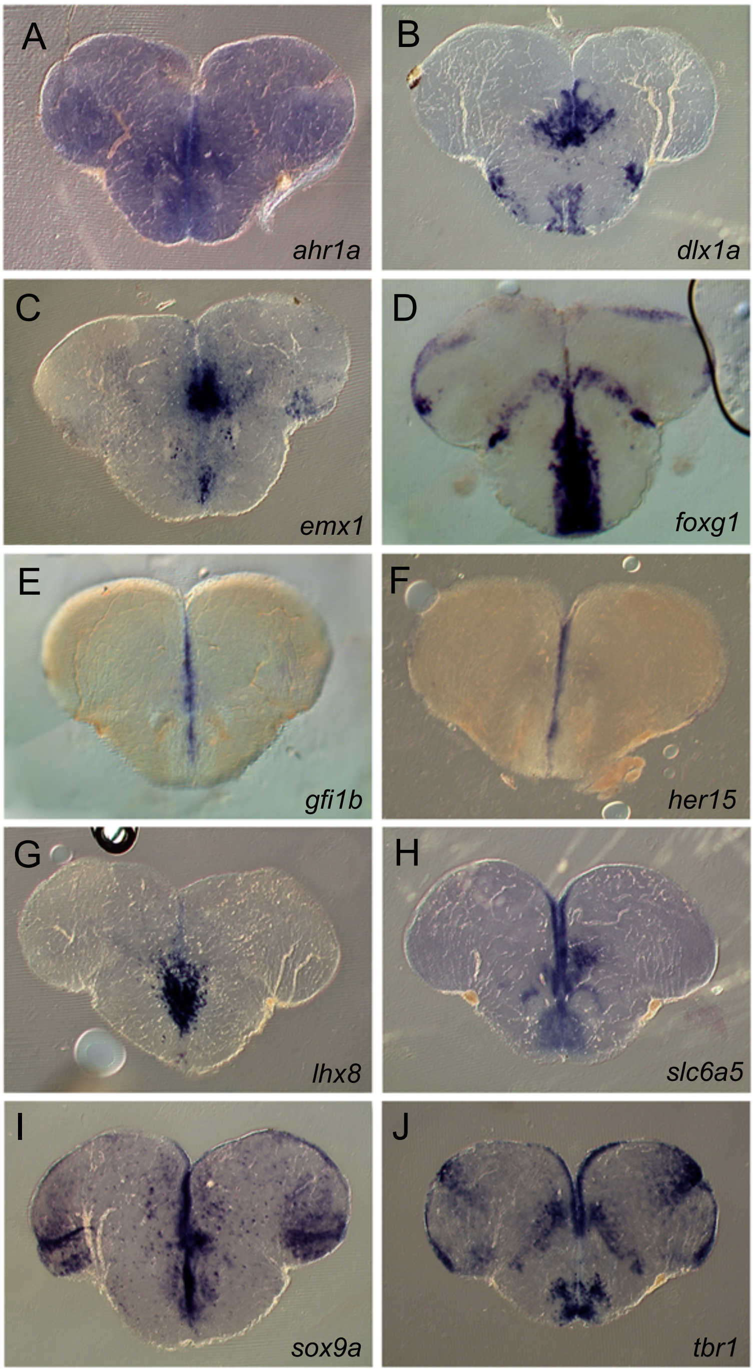

<u>Additi onal data file 6.</u> Expression in the adult brain of the 10 differentially regula ted transcripts ch osen for validati on (in situ hybridiz ation -blue signal- on cr oss sections at t elencephalic levels, dorsal up). *ahr1a* (A), *dlx1a* (B), *emx1* (C), *foxg1* (D), *gfi1b* (E), *her15* (F), *lhx8* (G), *slc6a5* (H), *sox9a* (I) and *tbr1* (J) are expressed in the adult zebrafish brain, including the telencephalon, as illustrated here. *gfi1b* and *her15* are restricted to the ventricular zone (midline, arrows). d=dorsal telenceph alic area (pallium); v=ventral telencephalic area (subpallium).
