# Supplementary material for: Zebrafish reward mutants reveal novel transcripts mediating the behavioral effects of amphetamine
Source: Genome Biol. 2009 Jul 31;10(7):R81. doi: 10.1186/gb-2009-10-7-r81 (PMC2728535; doi:10.1186/gb-2009-10-7-r81)
Supplement: Additional data file 8 — Expression of her15, gfi1b, foxg1 and lhx8 in the adult brain upon amphetamine administration. [file gb-2009-10-7-r81-S8.pdf]

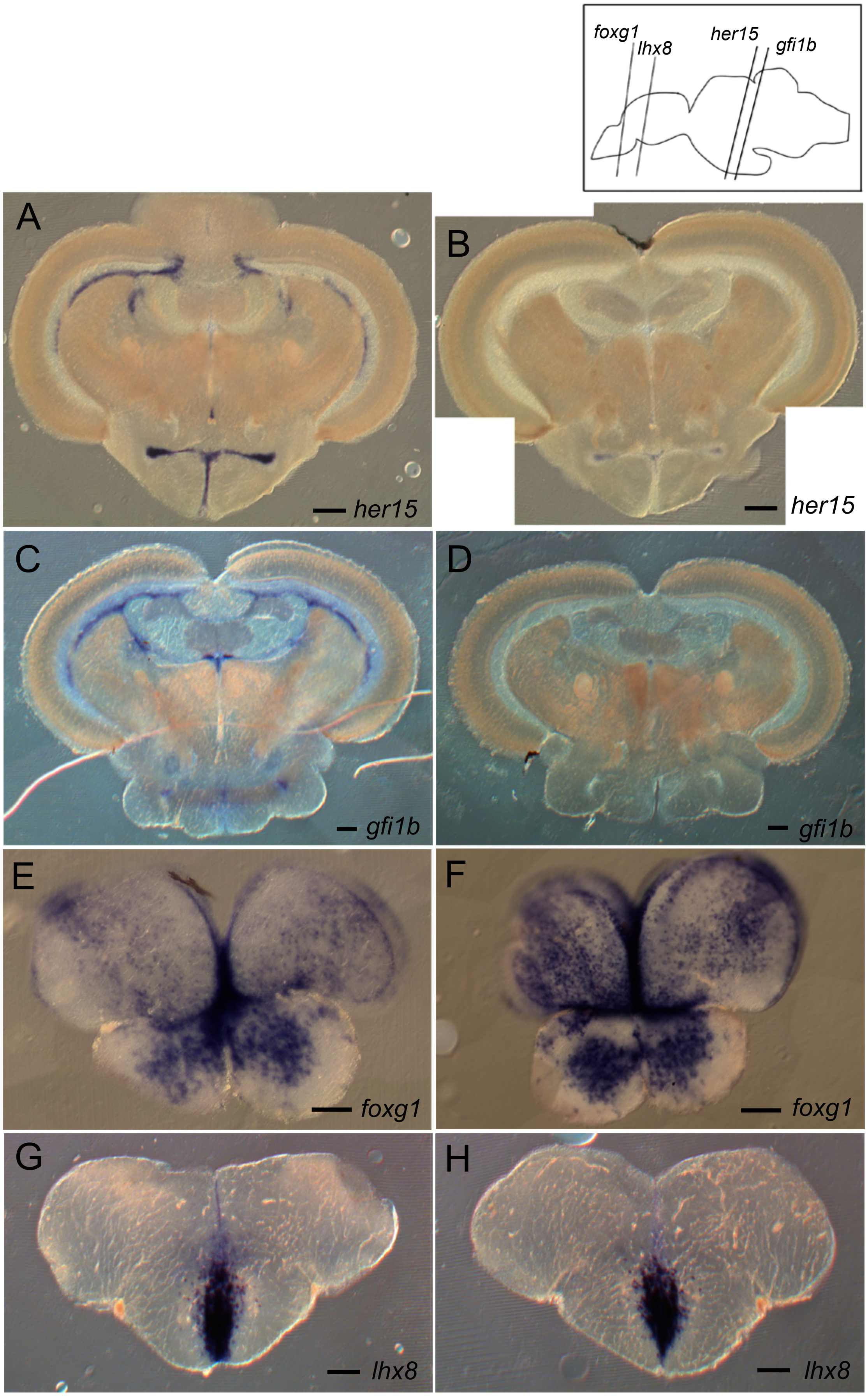

Additional data file 8. Expression of *her15*, *gfi1b*, *foxg1* and *lhx8* upon chronic amphetamine administration, as revealed by in situ hybridization. The expression of *her15* and *gfi1b* was lost upon amphetamine administration throughout the brain (A-D) (see also Fig.2A-D). The expression of *foxg1* and *lhx8* were changed in the parvocellular preoptic nucleus (*foxg1* and *lhx8*) and in the ventral zone of the periventricular hypothalamus (*foxg1*) (see Fig.2E-H) but not in other areas of the brain, including the telencephalon (E-H).
