# Supplementary material for: Zebrafish reward mutants reveal novel transcripts mediating the behavioral effects of amphetamine
Source: Genome Biol. 2009 Jul 31;10(7):R81. doi: 10.1186/gb-2009-10-7-r81 (PMC2728535; doi:10.1186/gb-2009-10-7-r81)
Supplement: Additional data file 9 — Primers and probes used for qPCR. [file gb-2009-10-7-r81-S9.ppt]

## Slide 1
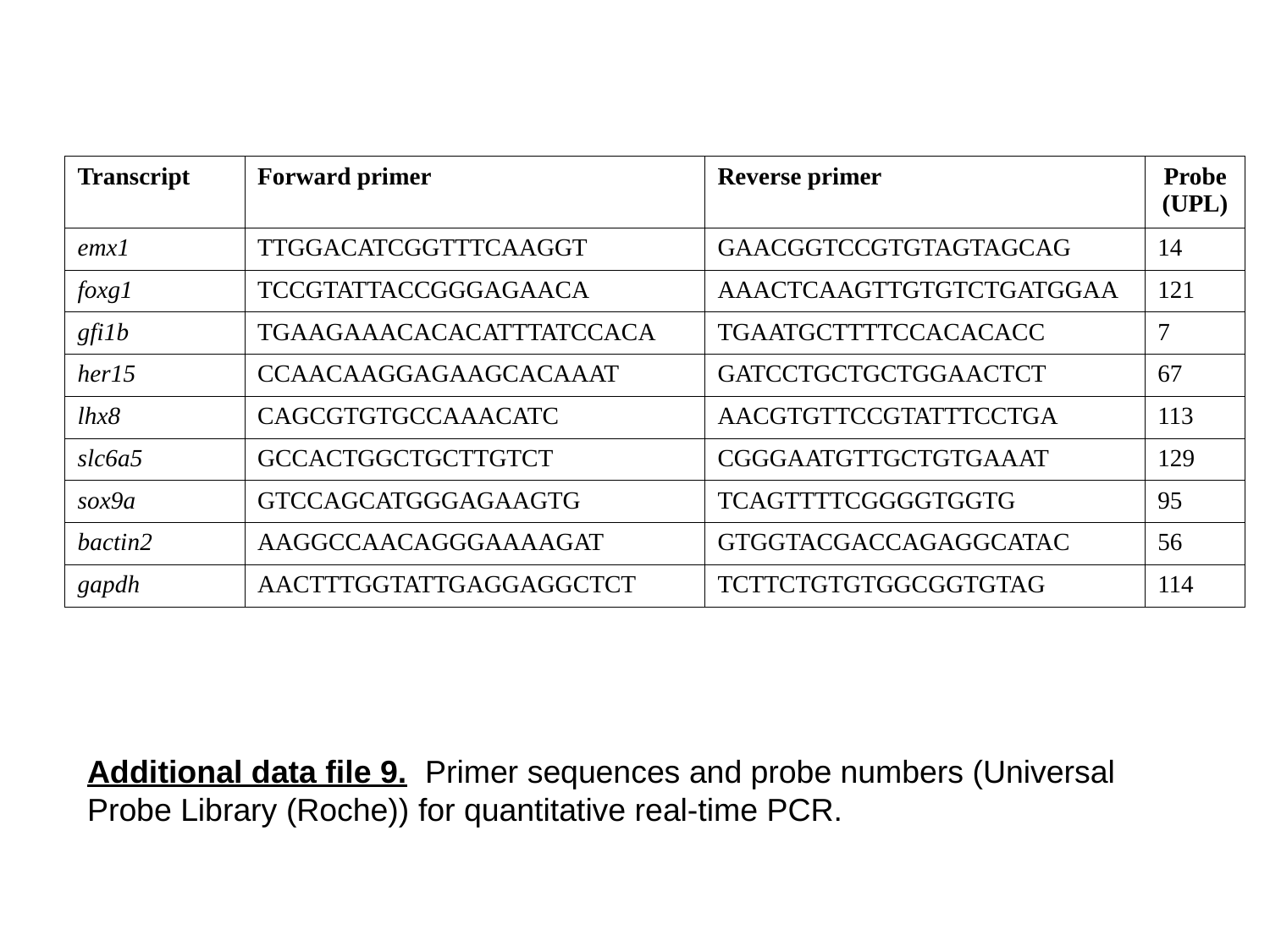

| Transcript | Forward primer | Reverse primer | Probe (UPL) |
| --- | --- | --- | --- |
| emx1 | TTGGACATCGGTTTCAAGGT | GAACGGTCCGTGTAGTAGCAG | 14 |
| foxg1 | TCCGTATTACCGGGAGAACA | AAACTCAAGTTGTGTCTGATGGAA | 121 |
| gfi1b | TGAAGAAACACACATTTATCCACA | TGAATGCTTTTCCACACACC | 7 |
| her15 | CCAACAAGGAGAAGCACAAAT | GATCCTGCTGCTGGAACTCT | 67 |
| lhx8 | CAGCGTGTGCCAAACATC | AACGTGTTCCGTATTTCCTGA | 113 |
| slc6a5 | GCCACTGGCTGCTTGTCT | CGGGAATGTTGCTGTGAAAT | 129 |
| sox9a | GTCCAGCATGGGAGAAGTG | TCAGTTTTCGGGGTGGTG | 95 |
| bactin2 | AAGGCCAACAGGGAAAAGAT | GTGGTACGACCAGAGGCATAC | 56 |
| gapdh | AACTTTGGTATTGAGGAGGCTCT | TCTTCTGTGTGGCGGTGTAG | 114 |
Additional data file 9. Primer sequences and probe numbers (Universal Probe Library (Roche)) for quantitative real-time PCR.
