# Supplementary material for: Zebrafish reward mutants reveal novel transcripts mediating the behavioral effects of amphetamine
Source: Genome Biol. 2009 Jul 31;10(7):R81. doi: 10.1186/gb-2009-10-7-r81 (PMC2728535; doi:10.1186/gb-2009-10-7-r81)
Supplement: Additional data file 10 — Primers used to clone in situ hybridization probes. [file gb-2009-10-7-r81-S10.ppt]

## Slide 1
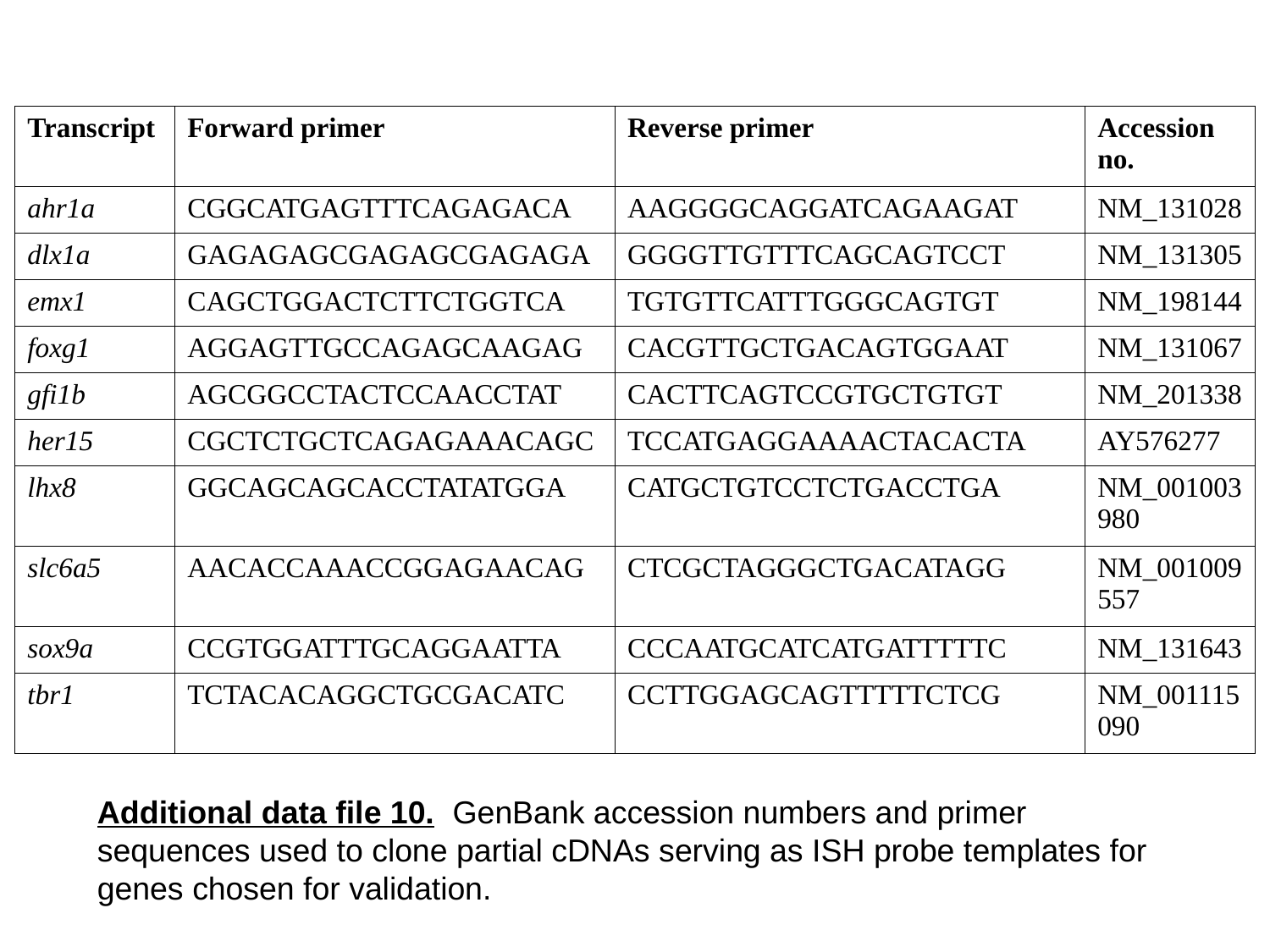

| Transcript | Forward primer | Reverse primer | Accession no. |
| --- | --- | --- | --- |
| ahr1a | CGGCATGAGTTTCAGAGACA | AAGGGGCAGGATCAGAAGAT | NM\_131028 |
| dlx1a | GAGAGAGCGAGAGCGAGAGA | GGGGTTGTTTCAGCAGTCCT | NM\_131305 |
| emx1 | CAGCTGGACTCTTCTGGTCA | TGTGTTCATTTGGGCAGTGT | NM\_198144 |
| foxg1 | AGGAGTTGCCAGAGCAAGAG | CACGTTGCTGACAGTGGAAT | NM\_131067 |
| gfi1b | AGCGGCCTACTCCAACCTAT | CACTTCAGTCCGTGCTGTGT | NM\_201338 |
| her15 | CGCTCTGCTCAGAGAAACAGC | TCCATGAGGAAAACTACACTA | AY576277 |
| lhx8 | GGCAGCAGCACCTATATGGA | CATGCTGTCCTCTGACCTGA | NM\_001003980 |
| slc6a5 | AACACCAAACCGGAGAACAG | CTCGCTAGGGCTGACATAGG | NM\_001009557 |
| sox9a | CCGTGGATTTGCAGGAATTA | CCCAATGCATCATGATTTTTC | NM\_131643 |
| tbr1 | TCTACACAGGCTGCGACATC | CCTTGGAGCAGTTTTTCTCG | NM\_001115090 |
Additional data file 10. GenBank accession numbers and primer sequences used to clone partial cDNAs serving as ISH probe templates for genes chosen for validation.
